# Vaginismus

**Published:** 1871-10

**Authors:** Theophilus Parvin


From The American Practitioner.

### VAGINISMUS.

BY THEOPHILUS PARVIN, M.D.

Spasmodic contraction of the vaginal sphincter is a disorder which has not until quite recently found a place in any of our text-books on diseases of women. As there were heroes before Agamemnon, so there were surgeons before our talented and famous countryman, Dr. Sims, presented his paper to the London Obstetrical Society, giving the name, the pathology and the therapeutics of the affection—surgeons who had previously traversed the same territory and arrived at the same ends.

Probably the first announcement made in regard to this disorder was by Dupuytren, in a clinical lecture* upon *fissure of the anus*, and was in these words: "This fissure, with consequent spasm, has not until the present been observed, except at the anus. M. Pinel Grandchamp has seen a similar case involving the vulva. The contraction had become so great that sexual intercourse was impossible. Convinced that the malady was analogous to fissure of the anus, M. Grandchamp made a deep incision, dividing for two inches the fourchette, the mucous membrane, and the constrictor of the vulva. The contraction closed, and the cure was complete."

Huguier, in his inaugural thesis, August, 1834, described the disease and its treatment.

Finally, without widening this retrospect,† vaginismus, as now termed, was made the subject of several communications in the *Bulletin Général de Thérapeutique Médicale et Chirurgicale* in the summer and autumn of 1861, while Dr. Sims' paper was presented to the Obstetrical Society in November of that year, the first of his cases having occurred in 1857. Michon, writing in August, 1861, narrates numerous cases, the first of which he was called to in 1847 by Chomel, who recognized the nature of the malady, and foresaw the surgical

*The volume in which this lecture is found was published in 1839; but the lecture was delivered some time before, as the author died in 1835. Upon referring to the Lancet, February 16, 1833, Dupuytren's lecture will be found much abridged, and containing no allusion to spasmodic contraction of the vagina.

†Professor Borelli, of Turin, published in 1851 a case of *spasm of the vulva*. After reciting the symptoms and treatment, he concludes that the affection was spasmodic rather than inflammatory, and of the same character as spasm of the anus; and that forced dilatation ought to succeed as well in this vulval as in anal spasm.

operation advisable for its relief. Nevertheless, to Dr. Sims belongs the honor of having, independently and entirely without knowledge of what others had done, worked out the problem of the disease and its therapeutics.

Some remarks made by Debout upon the etiology of this affection are so just that no apology need be offered for briefly presenting them. It is a general law of pathology, that when the mucous membrane covering a muscular layer becomes the seat of a prolonged and severe inflammation, the muscular fibres are thrown into spasmodic contraction. If the inflammation persists, permanent contraction may ensue—*e. g.*, as in the esophagus and urethra. When the muscular plane is a sphincter, as at the anus, the vulva, the eyelids, it often suffices that the mucous membrane may be the seat of any lesion which exalts its normal sensibility, as a hyperæsthesia, a fissure, etc. The manifestations of this law, notwithstanding their multiplicity and frequence, escaped the sagacity of observers until the illustrious Boyer pointed it out in his chapter upon fissure of the anus, as existing in reference to diseases of the mucous membrane, causing muscular spasm of the sphincter; and to him, too, belongs the honor of having pointed out, if not the best, the most powerful means of treatment—viz., incision of the contracted sphincter.

I shall now, in a few words, present three cases that occurred to me within the last twelve months.

Mrs. ——, fifty years of age, married, and the mother of a child some fifteen years of age, consulted me for a sensitive tumor at the mouth of the urethra, which had existed for a number of years. In this case vaginismus had gradually occurred, consequent upon the tumor, until sexual intercourse had become more and more painful, and finally was discontinued five years before I saw the patient. The vaginal orifice was so sensitive and so contracted that the introduction of the index finger was exceedingly painful and well-nigh impossible; and yet this lady was heroic and patient, and could endure pain and suffering quite as well as most of her sex. She was content with the removal of the urethral tumor, declining an operation to restore marital rights.

The second case occurred in a recently-married lady for five months a wife, during which repeated attempts at intercourse were fruitlessly made. The lady was young, the picture of health, well-formed and well-nourished, with no excessive development of the nervous system. Her general health was excellent, and there was no disease of the sexual organs, of the rectum, or of the bladder, to explain the vaginal spasm. It depended purely upon a local hyperæsthesia. In this case the treatment pursued was forced, then progressive dilatation. The dilators used were the glass instruments of Dr. Sims. Before introducing one, which was done daily, it was smeared with an ointment of simple cerate and atropia. The instrument was retained from twenty to thirty minutes. In two weeks complete cure.

The third case occurred in a lady who had been married some years, but who was childless from chronic endometritis. Consequent upon this endometritis there was more or less copious and constant purulent discharge from the uterus, producing great vaginal and vulval irritation and some erosions, with now and then actual vaginitis. For many months sexual intercourse had not been attempted. Here the vaginismus at times was so great that even the bulbous extremity of a self-injecting apparatus could not be introduced, and at any time the introduction of a cylindrical speculum of smallest size was exceedingly painful and difficult. The caliber of the vagina throughout seemed to be lessened; and such a condition is readily explicable by supposing an extension of inflammation to the connective tissue, with consequent hypertrophy and contraction.

Debout, in discussing this disorder, adopts as one of his conclusions, that it is most rational to commence by treating the lesion, if still existing, which has produced it; and when this lesion is cured, resort to dilatation. But it is manifest that such a practice would be utterly inapplicable in such a case as just narrated; for how reach the disease of the uterus when the very entrance of the avenue thereto was closed?

So, too, dilatation was out of the question; for the vaginal spasm and the vaginal contraction, with the irritable and sensitive condition of the entire vaginal surface, would render the process exceedingly slow and painful—possibly, even prevent its success. There remained, therefore, only operation by cutting, and this was done, and the subsequent treatment pursued as directed by Dr. Sims. (Obstetrical Transactions, vol. iii., page 364.) "Place the patient (fully chloroformed) as for lithotomy—on the back; pass the index and middle fingers of the left hand into the vagina; separate them laterally, so as to dilate the vagina as widely as possible, putting the fourchette on the stretch; then, with a common scalpel, make a deep cut through the vaginal tissue on the right of the mesial line, bringing it from above downward, and terminating at the raphé of the perineum. This cut forms one side of a Y. Then pass the knife again into the vagina, still dilating with the fingers as before, and cut in like manner on the opposite side from above downward, uniting the two incisions at or near the raphé, and prolonging them quite to the perineal integument. Each cut will be about two inches long—*i. e.*, half an inch or more above the edge of the sphincter, half an inch through its fibers, and an inch from its lower edge to the perineal raphé. Of course this will vary in different subjects, according to the development of tissue in each. To perfect the cure, it is necessary for the patient to wear for a time a properly-adapted bougie or dilator."

Dr. Sims does not introduce the dilator, unless there is much hemorrhage, until twenty-four hours after. It is worn two or three hours each morning and each afternoon. The cure is generally accomplished in two or three weeks.

The operation was successful; but the tendency to recurrence of the disorder was so great that the dilators had to be used daily for a much longer period than two or three weeks.

Here it may be well to refer to one of the dangers that may be met with in this operation—for Dr. Sims tells us that "in two instances the bleeding was excessive"—and to some of the different methods of operating that have been advised. And first of the latter. Huguier, operating in 1831 upon his first case, used a probe-pointed bistoury, introduced an inch and a half within the vagina, cutting within outward on one side, and then on the other, "dividing the opening of the vagina on either side to the extent of seven or eight lines." Considerable hemorrhage occurred in this case.

Michon, in cases where the hymen still remained, made with a probe-pointed bistoury three incisions, one medium and two lateral, through this structure, and extending to the sphincter, but not involving it. In other cases, double subcutaneous myotomy is made two centimeters distant on each side from the raphé, each division of the muscle being made from without toward the vagina, the extent and direction of the section regulated by the index finger of the left hand placed in the vagina.

Dr. Emmett's operation is done by passing the left index finger into the vagina, "elevating upon it the sphincter, which feels like a cord rolling upon it," then dividing with a pair of scissors the muscle upon each side of the perineal junction.

Burns' operation was division of the pubic nerve. Simpson's modification consisted in making this division subcutaneously. Dr. Savage believes a not uncommon form of vaginismus is due to the perineal body not having its usual elastic character, and is readily cured by "a median perpendicular incision three parts through it."

The instances where dangerous and even fatal hemorrhage has occurred from wounds of the lower portion of the vagina are not very numerous, but are explicable when we remember how freely the perineal and pelvic venous systems communicate, the absence of valves and the abundant supply of blood, and the existence of erectile plexuses. Occupying nearly three-fourths of either side of the vagina at its entrance is the *bulb* of the vagina, "an oblong body, about an inch and a half long, and nearly half an inch thick at the broader or lower end." Immediately below this is the vulvo vaginal gland, whose excretory duct usually opens at the junction of the inferior fourth with the superior three-fourths of the vaginal orifice, just outside of the hymen or of the lateral *carunculæ myrtiformes*, external to them and internal to the nymphæ. This opening may sometimes be recognized by its reddish border. Of course the nearer any deep incisions, made for the relief of vaginismus, approach the raphé, the less risk of hemorrhage; and on no account should they be made so far upward—the patient supposed to be lying on her back—as to run the risk of involving the bulb, or even the vulvo-vaginal gland. My own preference is, where a cutting operation is necessary, for comparatively superficial incisions, and then immediately forced dilatation with the index and medius of each hand, stretching apart the vaginal orifice.

Undoubtedly, the majority of cases of vaginismus can be cured without resorting to the knife. The removal of the cause is frequently sufficient in recent cases. The application of local sedatives may answer in others. Still others may be cured by dilatation, either *gradual*, as with glass bougies or gum-elastic bags distended with air or water, or *abrupt*, as performed in a manner similar to that originally proposed by Récamier for spasmodic contraction of the sphincter ani.

